# Error Analysis and Suppression of Rectangular-Pulse Binary Phase Modulation Technology in an Interferometric Fiber-Optic Sensor

**DOI:** 10.3390/s25154839

**Published:** 2025-08-06

**Authors:** Qian Cheng, Hong Ding, Xianglei Pan, Nan Chen, Wenxu Sun, Zhongjie Ren, Ke Cui

**Affiliations:** 1 School of Physical Science and Technology, Nantong University, Nantong 226019, China; m15061287152@163.com (Q.C.); 15205125123@163.com (H.D.); ntu_chennan@ntu.edu (N.C.); sunwenxu@ntu.edu.cn (W.S.); 2 School of Electronic and Optical Engineering, Nanjing University of Science and Technology, Nanjing 210094, China; pxl@njust.edu.cn (X.P.); njustcuik@njust.edu.cn (K.C.)

**Keywords:** fiber-optic sensor, interferometer, phase modulation, phase demodulation

## Abstract

In the field of interferometric fiber-optic sensing, the phase-shifting technique is well known as a highly efficient method for retrieving the phase signal from the interference light intensity. The rectangular-pulse binary phase modulation (RPBPM) method is a typical phase-shifting method with the advantages of high efficiency, low complexity, and easy array multiplexing. Exploring the impact of the parameters on the performance is of great significance for guiding its application in practical systems. In this study, the influence of the sampling interval and modulation depth deviation involved in the method is analyzed in detail. Through a comparative simulation analysis with the traditional heterodyne and phase-generated carrier methods, the superiority of the RPBPM method is effectively validated. Meanwhile, an improved method based on the ellipse fitting of the Lissajous figure is proposed to compensate for the error and improve the signal-to-noise-and-distortion ratio (SINAD) from 26.3 dB to 37.1 dB in a specific experiment. Finally, the experimental results guided by the above method show excellent performance in a practical vibration system.

## 1. Introduction

In recent decades, interferometric fiber-optic sensors (IFOSs) have been proven to measure various physical quantities such as acceleration, strain, and temperature [1,2,3,4,5,6,7,8,9,10]. Compared with the traditional electromagnetic sensor, the IFOS has the characteristics of high sensitivity, anti-electromagnetic interference, and strongly multiplexing capabilities, and has been widely used in perimeter monitoring, seismic wave detection, structural health monitoring, and other fields [11,12,13,14,15,16].

The IFOS takes the optical fiber as the transmission medium and obtains the corresponding physical quantity through the phase change in the optical fiber. The commonly used interferometers include the fiber Mach–Zehnder interferometer (MZI), fiber Michelson interferometer (MI), fiber Sagnac interferometer (SI), and fiber Fabry–Perot interferometer (FPI). For the above interferometers, the phase extraction technique for obtaining the final-phase information is crucially important. Therefore, several different interrogation methods have been investigated, such as 3 × 3 coupling multi-phase detection [17,18] and the phase-generator carrier (PGC) homodyne method [19,20,21,22]. Among these, as a typical phase-shifting method, the rectangular-pulse binary phase modulation (RPBPM) method has the characteristics of high precision, strong noise suppression ability, and low complexity, making it suitable for application systems [23]. Meanwhile, in [23], we proposed a Low-pass-filter (LPF)-assisted RPBPM method, which can effectively suppress DC and amplitude noise in the full frequency domain. Specifically, a simple rectangular pulse is applied to generate the [0, pi/2] phase-shifting modulation. With the help of an unbalanced interferometer, three-step [pi/2, 0, −pi/2] phase shifts can be generated, and the orthogonal signals can be collected. Then, the phase information can be obtained according to the equidistant sampling interval in a sampling window. To date, this method has been well promoted and applied. However, no relevant error and performance analysis of this method has been conducted so far to guide its applications. In fact, the existence of sampling interval and the deviation of modulation depth affect the signal demodulation [24]. These two are the main sources of error. In view of this, we systematically analyze the sampling interval and modulation depth involved in the system to explore its influences on the phase results. The sampling interval causes errors in the amplitude and frequency of the demodulated phase signal. Therefore, the sampling interval should be adjusted based on different conditions to improve the adaptability of the system. The modulation depth deviation also causes signal aberration [25]. To solve this problem, we propose an improved method based on the ellipse fitting of the Lissajous figure, which effectively improves the phase detection performance. The above work can effectively guide the application based on this method in the actual system and improve its performance to a certain extent.

## 2. Principle of the System

**Figure 1:**
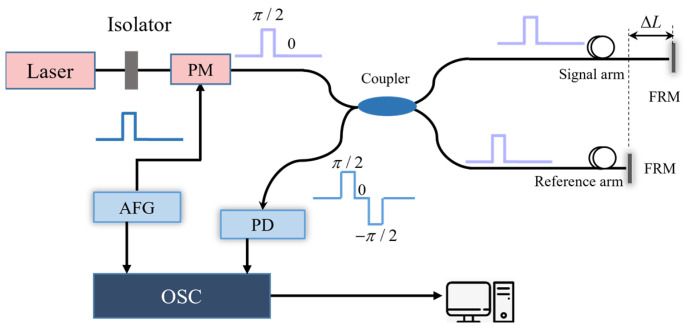
The schematic diagram of the RPBPM method.

The system is mainly composed of an unbalanced Michelson interferometer. The core generates the [0, pi/2] phase-shifting modulation for the laser source using a function generator. As shown in Figure 1, the modulated continuous laser transfers through the 2 × 2 coupler into the two arms of the interferometer. The returned interference signal contains three-step [pi/2, 0, −pi/2] phase shifts. A photodetector is responsible for receiving the interference signal and transmitting it to the oscilloscope (OSC). The OSC also receives the trigger reference signal generated by an arbitrary function generator (AFG) for sampling. The demodulated phase signals are displayed on the PC end.

**Figure 2:**
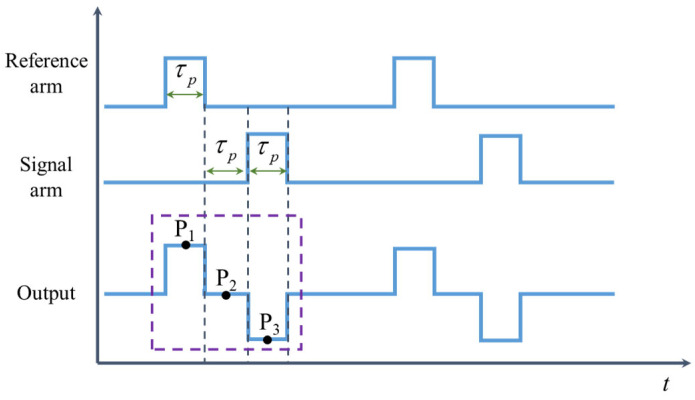
The timing relationship of the signals returned from the reference arm and the signal arm.

The generated phase-shifting modulation signal can be expressed as follows [23]:

(1) $$\varphi_{m}(t)=\left\{\begin{array}{l} \pi /2,\;kT\leq t<\tau_{p}+kT\\ 0,\;\tau_{p}+kT\leq t<(k+1)T \end{array}\right.$$

where *τ_p_* denotes the pulse width, and *T* denotes the cycle. The timing relationship of the signals returned from the reference arm and the signal arm is shown in Figure 2. There is a certain delay between the signals returned from the reference arm and the signal arm, which can be expressed as

(2) Δt=2nΔL/c

where *n* represents the refractive index, Δ*L* represents the length difference between the two arms, and *c* represents the speed of light. In our configuration, Δ*t* strictly equals to 2*τ_p_*. Then, the light intensity signal with three-step phase shifts is generated, where the phase shifts corresponding to P_1_, P_2_, and P_3_ are [pi/2, 0, −pi/2], respectively.

The specific light intensities corresponding to P_1_, P_2_, and P_3_ can be expressed as follows [23]:

(3) $$\begin{array}{l} I_{1}(t)=A+B\cos(\varphi (t)+\pi /2)\\ I_{2}(t)=A+B\cos(\varphi (t))\\ I_{3}(t)=A+B\cos(\varphi (t)-\pi /2) \end{array}$$

Then, a pair of orthogonal terms are calculated as follows:

(4) $$\begin{array}{l} I_{\cos}(t)=I_{3}(t)-I_{1}(t)\\ I_{\sin}(t)=2I_{2}(t)-(I_{1}(t)+I_{3}(t)) \end{array}$$

Finally, the phase can be obtained by the arc-tangent method:

(5) $$\varphi (t)=\tan^{-1}[\frac{I_{\cos}(t)}{I_{\sin}(t)}]$$

From the analysis, it can be clearly inferred that the phase can be effectively obtained. Furthermore, the slow fluctuations of parameters A and B do not interfere with the demodulation process described in the equation above [23].

The experimental configuration must satisfy the following two criteria: (1) it must be implemented using an unbalanced Michelson interferometer, and (2) the optical path difference in the unbalanced interferometer corresponds to a time delay of twice the modulated pulse width. In the practical applications, the time delay should be at least equal to the modulated pulse width.

## 3. Error Analysis

From the analysis of the principle above, it is evident that there exists a sampling interval Δ*t* between the three sampling points P_1_, P_2_ and P_3_, which is ignored in Equation (3). This sampling interval may lead to errors in the demodulation results. Meanwhile, the modulation depth of π/2 in Equation (3) may lead to depth deviation in practical applications, thereby causing demodulation distortion. This section focuses on analyzing the noise associated with these two factors and provides corresponding improvement recommendations based on the results.

### 3.1. The Error Caused by the Sampling Interval

From the analysis of the above principle, it is evident that there exists a sampling interval Δ*t* between the three sampling points P_1_, P_2_, and P_3_. This sampling interval may lead to errors in the demodulation results.

Considering the sampling interval Δ*t*, Equation (3) can be changed as follows:

(6) $$\begin{array}{l} I_{1}=A+B\cos(\theta (t-\Delta t)+\pi /2)\\ I_{2}=A+B\cos\theta (t)\\ I_{3}=A+B\cos(\theta (t+\Delta t)-\pi /2) \end{array}$$

According to the original arc-tangent algorithm, θ(t) can be expressed as follows:

(7) $$\begin{array}{l} \theta (t)={tan}^{-1}(\frac{I_{3}-I_{1}}{2I_{2}-(I_{1}+I_{3})})\\ \;={tan}^{-1}(\frac{\sin\theta (t+\Delta t)+\sin\theta (t-\Delta t)}{2\cos\theta (t)-(\sin\theta (t+\Delta t)-\sin\theta (t-\Delta t))}) \end{array}$$

**Figure 3:**
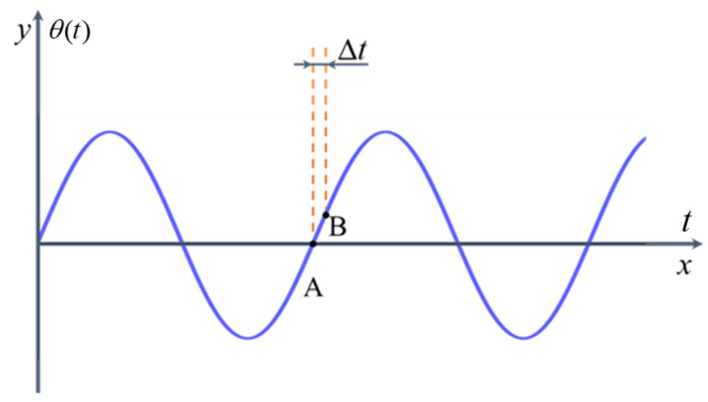
The vibration curve of θ(t).

It is evident that there exists a phase error in θ(t) caused by Δ*t*. Assuming θ(t)=Dsin(2πft), the corresponding vibration curve is shown in Figure 3. It shows that the value of θ(t) has the largest change rate at the position θ(t)=0. Therefore, the deviation caused by the sampling interval at point A is the largest. Meanwhile, the change in the vibration signal is approximately linear at point A. Considering this condition with the maximum deviation at point A, θ(t+Δt) and θ(t−Δt) can be given by

(8) $$\begin{array}{l} \theta (t+\Delta t)=D\sin(2\pi f(t+\Delta t))\approx D\sin(2\pi ft)+2\pi Df\Delta t\\ \theta (t-\Delta t)=D\sin(2\pi f(t+\Delta t))\approx D\sin(2\pi ft)-2\pi Df\Delta t \end{array}$$

Then, we have

(9) $$\begin{array}{l} \sin\theta (t+\Delta t)+\sin\theta (t-\Delta t)=\sin[D\sin(2\pi ft)+2\pi f\Delta t]+\sin[D\sin(2\pi ft)-2\pi f\Delta t]\\ \;=2\sin[D\sin(2\pi ft)]\cdot \cos2\pi Df\Delta t\\ 2\cos\theta (t)-(\sin\theta (t+\Delta t)-\sin\theta (t-\Delta t))\\ \;=2\cos[\sin(2\pi ft)]-2\cos[\sin(2\pi ft)]\cdot \sin(2\pi f\Delta t)\\ \;=2\cos[\sin(2\pi ft)](1-\sin(2\pi f\Delta t)) \end{array}$$

Substituting Equation (9) into Equation (7), θ(t) can be derived as follows:

(10) $$\begin{array}{l} \theta (t)={tan}^{-1}(\frac{\sin[D\sin(2\pi ft)]\cdot \cos2\pi Df\Delta t}{\cos[D\sin(2\pi ft)](1-\sin(2\pi Df\Delta t))})\\ \;={tan}^{-1}(\frac{\sin[D\sin(2\pi ft)]}{\cos[D\sin(2\pi ft)]}\cdot \frac{\cos2\pi Df\Delta t}{1-\sin(2\pi Df\Delta t)}) \end{array}$$

According to the above equation, *D*, *f*, and the sampling interval Δ*t* itself cause the phase demodulation error.

Supposing $g(D,f,\Delta t)=\frac{\cos2\pi Df\Delta t}{1-\sin(2\pi Df\Delta t)}$ and x=2πDfΔt, g(D,f,Δt) can be changed as

(11) $$g(x)=\frac{1}{2}\cdot \frac{2-x^{2}}{1-x}$$

Thus, we calculate the derivative of g(x):

(12) $$g^{,}(x)=\frac{1}{2}\cdot \frac{{\left(1-x\right)}^{2}+1}{{\left(1-x\right)}^{2}}=\frac{1}{2}\cdot [1+\frac{1}{{\left(1-x\right)}^{2}}]$$

According to Equation (12), constantly $g^{,}(x)\geq 1$, and we can define that the increase in *D*, and *f* and Δ*t* evidently increase the demodulated phase error.

### 3.2. The Error Caused by the Modulation Depth Deviation

Considering the modulation depth deviation δ, Equation (3) can be changed as follows:

(13) $$\begin{array}{l} I_{1}=A+B\cos(\theta (t)+\pi /2+\delta )\\ I_{2}=A+B\cos\theta (t)\\ I_{3}=A+B\cos(\theta (t)-\pi /2-\delta ) \end{array}$$

Thus, θ(t) can be derived as follows:

(14) $$\begin{array}{l} \theta (t)=\tan^{-1}(\frac{I_{3}-I_{1}}{2I_{2}-(I_{1}+I_{3})})=\tan^{-1}(\frac{\sin[\theta (t)-\delta ]+\sin[\theta (t)+\delta ]}{2\cos\theta (t)-\sin[\theta (t)-\delta ]+\sin[\theta (t)+\delta ]})\\ \;=\tan^{-1}(\frac{\sin(\theta (t))}{\cos(\theta (t))}.\frac{\cos\delta }{1+\sin\delta }) \end{array}$$

Assuming $h(\delta )=\frac{\cos\delta }{1+\sin\delta }$, we calculate the derivative of h(δ):

(15) $$h^{\prime }(\delta )=-\frac{1}{1+\sin\delta },\;0\leq \delta <\frac{\pi }{2}$$

where constantly $h^{\prime }(\delta )<0$; then, we can assume that the increase in δ causes an evidently higher demodulated phase error.

### 3.3. Simulation Analysis

**Figure 4:**
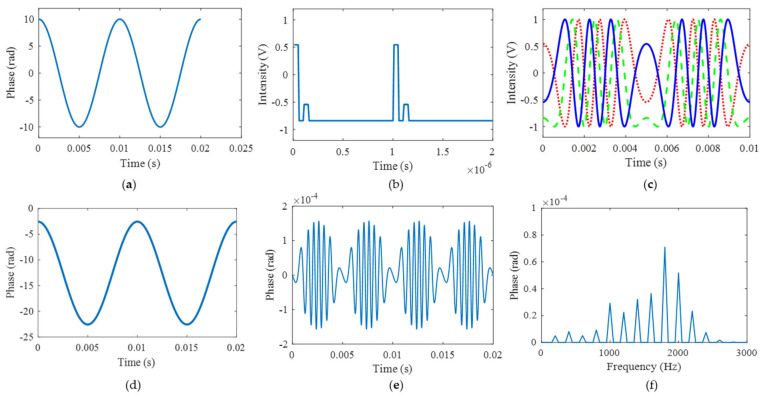
The simulation results of the sampling interval error: (**a**) the theoretical vibration signal; (**b**) the modulated interference signal; (**c**) the obtained three light intensities; (**d**) the final demodulated phase; (**e**) the time domain error; and (**f**) the frequency domain error.

To demonstrate our theory, simulation analysis is carried out in this part. Firstly, the sampling interval error is analyzed. The parameter settings are listed in Table 1. The theoretical vibration signal and the modulated interference light intensity are shown in Figure 4a,b. The interference light intensity has three-step [pi/2, 0, −pi/2] phase shifts in one modulation period, which is consistent with the parameter setting. Thus, the obtained three light intensities are shown in Figure 4c, where the phases satisfy the relationship of [pi/2, 0, −pi/2]. The final demodulated phase using the arc-tangent algorithm is shown in Figure 4d. The demodulated phase is consistent with the simulated vibration signal. By comparing the simulated vibration signal and demodulated phase, the time and frequency domain errors are depicted in Figure 4e,f. The maximum time domain error is about 1.56 × 10^−4^ rad, and the maximum frequency domain error is about 7.1 × 10^−5^ rad. The result shows that the error has little influence on the demodulation results. In order to quantitatively evaluate the demodulation performance, we define the demodulation criteria as follows: (1) the Relative Amplitude Distortion (RAD) is less than 1%; (2) the Minimum Harmonic Distortion (MHD) is more than 40 dB; and (3) the signal-to-noise distortion ratio (SINAD) is at least 30 dB. The RAD, MHR, SINAD here are 0.00000021%, 100.01 dB, and 95.09 dB, respectively, which shows good performance.

To further illustrate the influence of *D*, *f*, and Δ*t*, more detailed experiments have been carried out.

**Figure 5:**
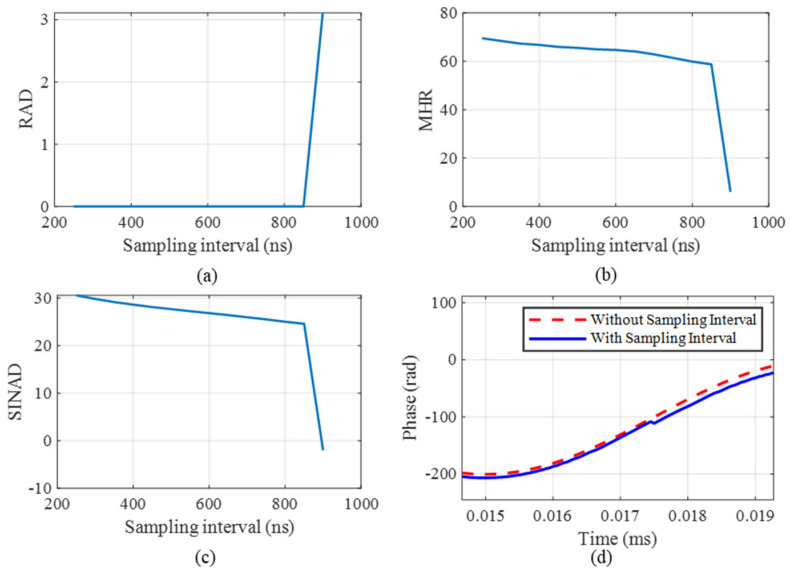
The error results with changes in the RAD, MHR, and SINAD. (**a**) The RAD, (**b**) MHR, (**c**) SINAD, and (**d**) comparison of the original signal with the demodulated signal.

With the sampling rate set to 200 kHz, signal frequency to 1 kHz, and amplitude to 100 rad, multiple modulated signals are generated for each sampling interval, ranging from 250 ns to 900 ns. Figure 5a–c depict the RAD, MHR, and SINAD results with different sampling intervals. By calculating the RAD, MHR, and SINAD, the demodulation signal is found to be distorted at 900 ns. Figure 5d shows the comparison results with and without sampling intervals at 900 ns. In detail, the red line represents the phase using the ideal demodulation without a sampling interval, and the blue line represents the result using the RPBPM method with a sampling interval. The comparison result indicates that the sampling interval has led to the distortion of the result.

**Figure 6:**
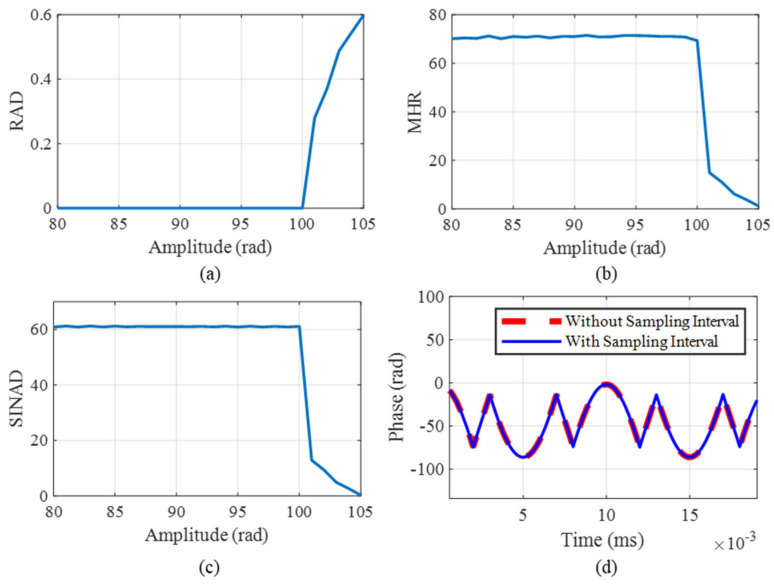
The error results with changes in the RAD, MHR, and SINAD. (**a**) The RAD, (**b**) MHR, (**c**) SINAD, and (**d**) comparison of the original signal with the demodulated signal.

With the sampling rate set to 200 kHz, vibration frequency to 1 kHz, and sampling interval to 250 ns, multiple modulated signals are generated for each amplitude, ranging from 80 to 105 rad. Figure 6a–c depict the RAD, MHR, and SINAD results with different amplitudes. The results indicate that, as the amplitude increases, harmonic interference in the demodulated signal becomes more pronounced. Additionally, the SINAD remains relatively high, but below 100 rad, suggesting good signal quality. The demodulation signal is found to be distorted at 101 rad. In order to further analyze the performance of the demodulation algorithm, we compare the result of the ideal demodulation without a sampling interval with that of the RPBPM method with a sampling interval. As illustrated in Figure 6d, the experimental results demonstrate that the distortion originates from the insufficient satisfaction of the sampling theorem, rather than being attributable to the proposed RPBPM method.

**Figure 7:**
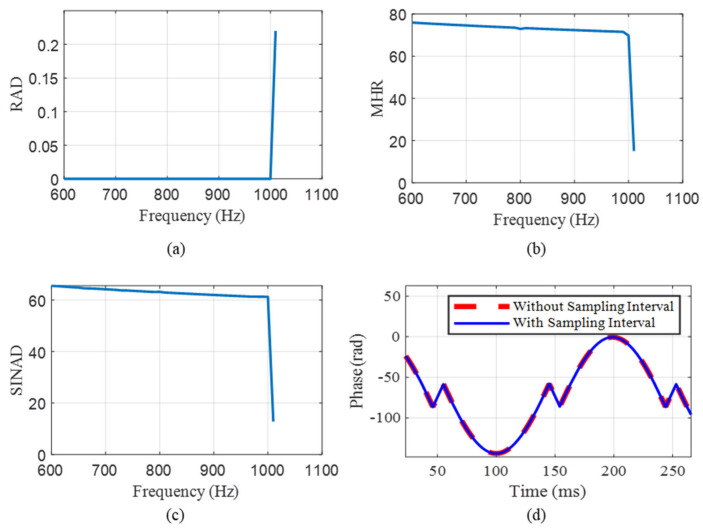
The error results with changes in the RAD, MHR, and SINAD. (**a**) The RAD, (**b**) MHR, (**c**) SINAD, and (**d**) comparison of the original signal with the demodulated signal.

With the sampling rate set to 200 kHz, vibration amplitude to 100 rad, and the sampling interval to 250 ns, multiple modulated signals are generated for each vibration frequency, ranging from 600 to 1001 Hz. Figure 7a–c depict the RAD, MHR, and SINAD results. The demodulation signal is found to be distorted at 1001 Hz. In order to further analyze the performance of the demodulation algorithm, we compare the result of the ideal demodulation without a sampling interval with that of the RPBPM method with a sampling interval. As illustrated in Figure 7d, the experimental results demonstrate that the distortion originates from the insufficient satisfaction of the sampling theorem, rather than being attributable to the proposed RPBPM method.

**Figure 8:**
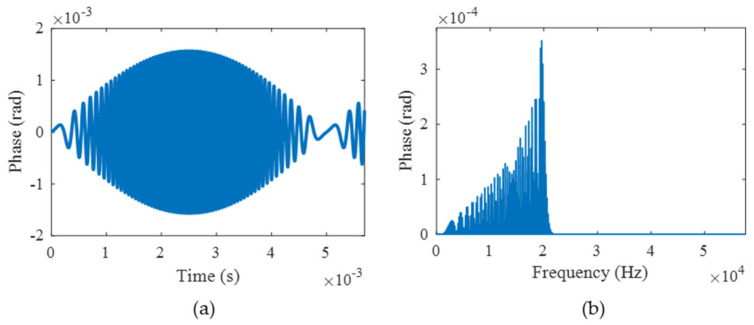
The error results when Δ*t* = 50 ns, *f* = 1000 Hz, and *D* = 100 rad. (**a**) The time domain error; (**b**) the frequency domain error.

The preceding analysis reveals that the principal cause of demodulation distortion originates from the sampling interval. By selecting appropriate sampling intervals in accordance with specific application requirements, this method demonstrates remarkable adaptability across practical implementations. Finally, assuming Δ*t* = 50 ns, *f* = 1000 Hz, and *D* = 100 rad, as shown in Figure 8, the time and frequency errors are 0.0016 rad and 0.000353 rad, respectively. The RAD, MHR, and SINAD are 0.0000782%, 85.5 dB, and 75.1 dB. The results evidently accord with the lossless standard. Hence, by setting the sampling interval reasonably, the vibration signal with an upper-limit amplitude of 100 rad and an upper-limit frequency of 1000 Hz can be effectively achieved.

**Figure 9:**
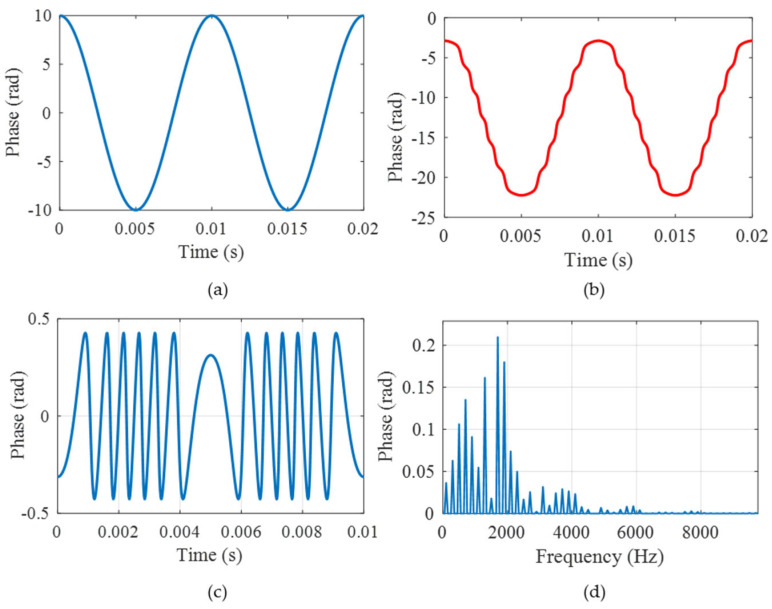
The simulation results of the modulation depth deviation: (**a**) the theoretical vibration signal; (**b**) the demodulated phase; (**c**) the time domain error; and (**d**) the frequency domain error.

Secondly, the error simulation caused by the modulation depth deviation was carried out. The parameter settings are listed in Table 2. The theoretical vibration signal is shown in Figure 9a, and the demodulated phase is shown in Figure 9b. Comparing the two curves, we observe that the demodulated phase has an obvious distortion. As shown in Figure 9c,d, the time and frequency errors are 0.427 rad and 0.210 rad, respectively, and we obtain the RAD, MHR, and SINAD, which are 3.1285%, 33.542 dB, and 27.9183 dB, respectively, resulting in the serious distortion of phase demodulation. Thus, the modulation depth deviation error needs to be compensated for.

## 4. Comparison with Other Methods

After the simulation, we conducted a comparative analysis among our RPBPM method, the PGC method, and the heterodyne method. The sampling frequency is set to 100 kHz, and the vibration amplitude is set to 1 rad, and the demodulation performances of the three methods at different vibration frequencies are shown in Table 3, Table 4 and Table 5. The PGC and heterodyne demodulation do not meet the demodulation criteria at 3000 Hz. Compared to the PGC and heterodyne demodulation techniques, the RPBPM method exhibits superior performance for bandwidth detection.

Next, the sampling frequency is set to 100 kHz, and the vibration frequency is set to 1000 Hz, and the demodulation performances of the three methods at different vibration amplitudes are shown in Table 6, Table 7 and Table 8. The result shows that the error of the PGC method increases at 10 rad and the error of heterodyne demodulation increases at 20 rad. The SINAD of the RPBPM method remains about 61 dB within the range of 20–100 rad, and the RAD error is close to 0%, showing extremely high amplitude accuracy. The demodulation performance drops sharply at 101 rad. It is clear from the previous simulations that the distortion originates from the insufficient satisfaction of the sampling theorem. The above comparative simulations demonstrate the superiority of our proposed RPBPM method.

## 5. Suppression of Modulation Depth Deviation

We can reasonably set the sampling interval according to the application requirements to meet the demodulation criteria, but the modulation depth deviation error needs to be compensated. Regarding this issue, this section, according to the above theoretical and simulation analyses, we achieve the error calibration by fitting the ellipse of the Lissajous figure. The detailed steps are as follows:

Step 1: obtain the three-step phase-shifting interference signals and calculate original orthogonal terms.

Step 2: plot the Lissajous figure using the original orthogonal terms.

Step 3: obtain the elliptic fitting of Lissajous figure and calculate the calibration coefficient.

Step 4: calibrate the orthogonal term using the calibration coefficient and obtain the calibrated orthogonal terms.

Step 5: achieve the final-phase demodulation using the calibrated orthogonal terms.

**Figure 10:**
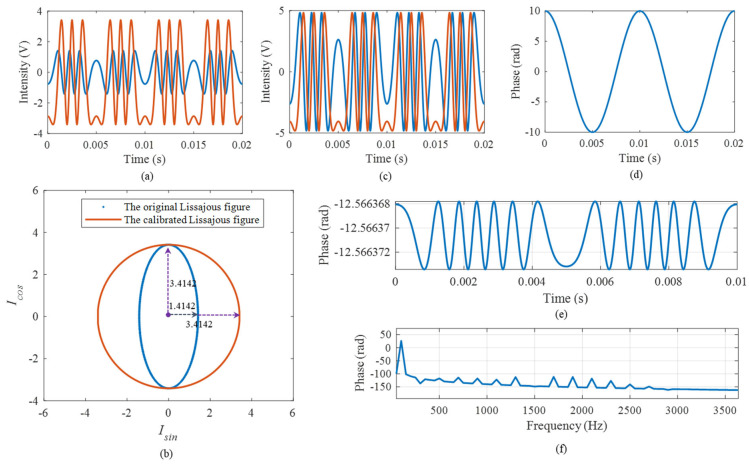
The suppression of modulation depth deviation: (**a**) the two original orthogonal signals; (**b**) the original and calibrated orthogonal Lissajous figure; (**c**) the calibrated orthogonal signals; (**d**) the demodulated result; (**e**) the time domain error; (**f**) the frequency result.

Considering the above simulation as an example, the two original orthogonal signals obtained according to the three-step phase-shifting light intensity are shown in the Figure 10a and the corresponding Lissajous figure is shown by the blue dots in Figure 10b. It shows that the two signals do not satisfy the orthogonal relationship. We performed ellipse fitting on the curve and then obtained the long axis x = 3.4142 and the short axis y = 1.4142, respectively. Thus, we obtained the calibration factor. The red line in Figure 10b shows the calibrated Lissajous figure and the calibrated orthogonal signals are shown in Figure 10d, where the two orthogonal terms satisfy the orthogonal relationship. The demodulated phase, the time-domain error and the frequency result are shown in Figure 10d–f. Here, after calibration, we can see that the error can be ignored successfully.

## 6. Standard Vibration Experiments

First, the standard vibration experiments using a piezoelectric transducer (PZT) in our laboratory were carried out. A homemade narrow-linewidth laser is employed in our experiments, which works at 1550 nm with a 1 KHz linewidth. Meanwhile, we use a phase modulator to generate high-frequency rectangular pulses. The length difference between the two arms of the Michelson interferometer is 5 m. We placed a PZT to generate the standard vibration.

**Figure 11:**
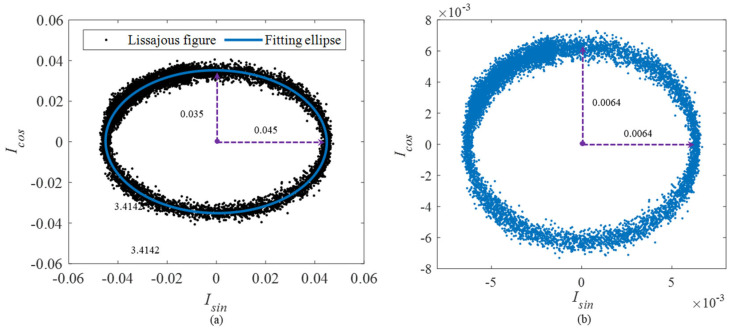
The Lissajous figures before and after the calibration: (**a**) the original Lissajous figure and (**b**) the calibrated Lissajous figure.

**Figure 12:**
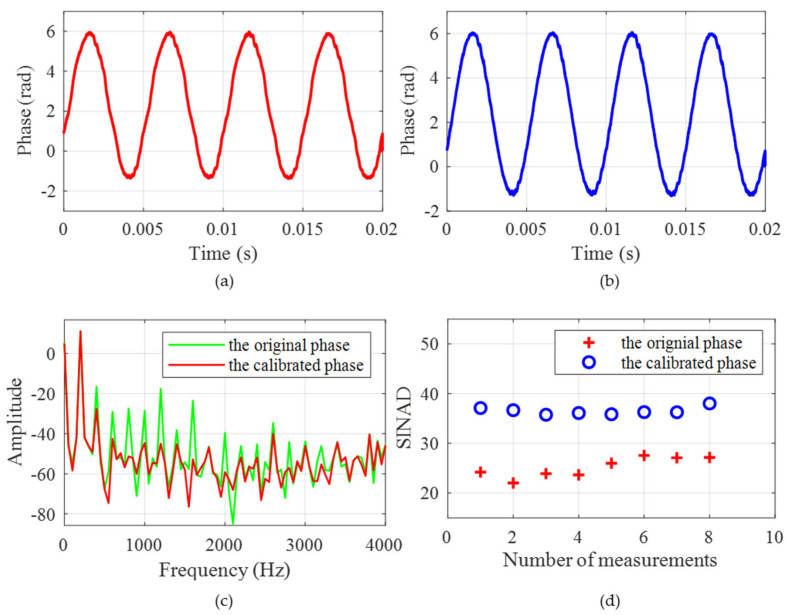
The experiment for modulation depth deviation: (**a**) the phase result before calibration; (**b**) the phase result after calibration; (**c**) the frequency domain results; and (**d**) multiple results of phase demodulation.

Adjusting the modulation voltage of the phase modulator, the three-step phase-shifting signals are obtained. The corresponding Lissajous figure is shown in Figure 11a. The fitting ellipse is not a strict circle where the long axis x = 0.045 and the short axis y = 0.035, which means the two orthogonal signals are not strictly orthogonal. The calibration coefficient is obtained by using the fitting ellipse. The calibrated Lissajous figure is shown in Figure 11b, where the long axis x = 0.0064 and the short axis y = 0.0064. The orthogonal relationship is basically satisfied at this time. The phase results before and after the calibration are shown in Figure 12. It shows that the errors demodulated by the original orthogonal signals is significantly higher than that using the calibrated signals. Specifically, the SINADs are 26.3 dB and 37.1 dB, respectively. Obviously, the result using the calibrated orthogonal signals has a better SINAD, which satisfy the demodulation criteria. Through the comparative analysis, the effectiveness of calibration is verified.

**Figure 13:**
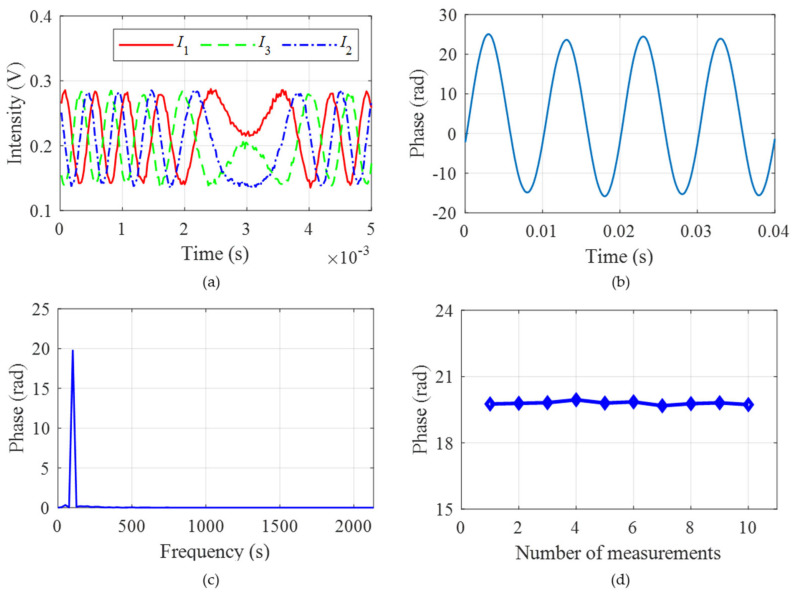
Further comprehensive experiments: (**a**) the calibrated three-step light intensity; (**b**) the demodulated phase result; (**c**) the frequency domain result; and (**d**) multiple results of phase demodulation.

After completing the analysis of sampling interval and achieving the suppression of modulation error, further comprehensive experiments are carried out. The sampling interval is set to 25 ns, and the PZT is used to generate a vibration signal with a frequency of 100 Hz. As shown in Figure 13a, the calibrated three-step light intensity signals satisfy the phase relationship of [pi/2, 0, −pi/2]. The phase result shown in Figure 13b is calculated using the three-step phase-shifting algorithm. It is evident that the signal recovered well. The frequency curve obtained by FFT transformation is shown in Figure 13c. The result shows that the signal frequency is 100 Hz, which is consistent with that generated by the PZT. Multiple results of the phase demodulation are shown in Figure 13d, which prove the stability of this method.

## 7. Experiments in a Practical Vibration System

**Figure 14:**
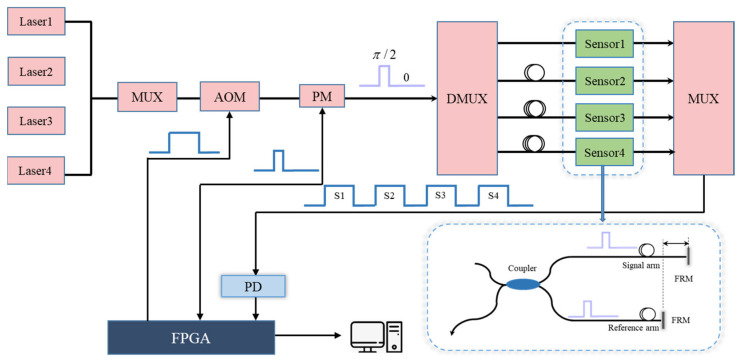
The practical vibration system structure diagram.

Finally, we carried out the practical vibration experiments based on the time division multiplexing (TDM)/wavelength division multiplexing (WDM) technology. As shown in Figure 14, four hybrid lasers with different wavelengths were mixed to obtain the light source. The laser source was then modulated by an acousto-optic modulator (AOM, produced by Gooch & Housego Inc. Ilminster, UK) to generate the light pulses. After phase modulation, the light pulses entered the corresponding sensor using a Demuxer. Each sensor corresponded to a specific wavelength and time interval. The return signals carrying the sensing information were mixed and transferred into the PD. The field programmable gate array (FPGA) received the signals and transmitted the extracted light intensities to the PC end.

**Figure 15:**
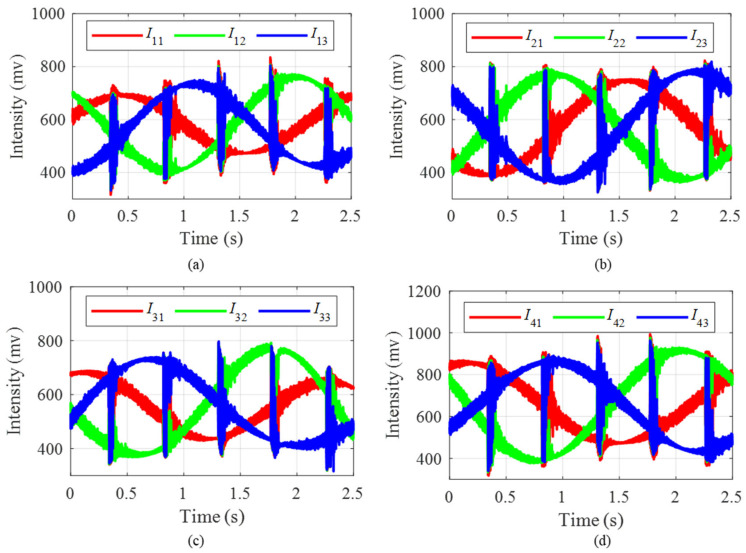
The three-step light intensity signals corresponding to the four sensors: (**a**) three-step light intensity signals corresponding to S1; (**b**) three-step light intensity signals corresponding to S2; (**c**) three-step light intensity signals corresponding to S3; (**d**) three-step light intensity signals corresponding to S4.

**Figure 16:**
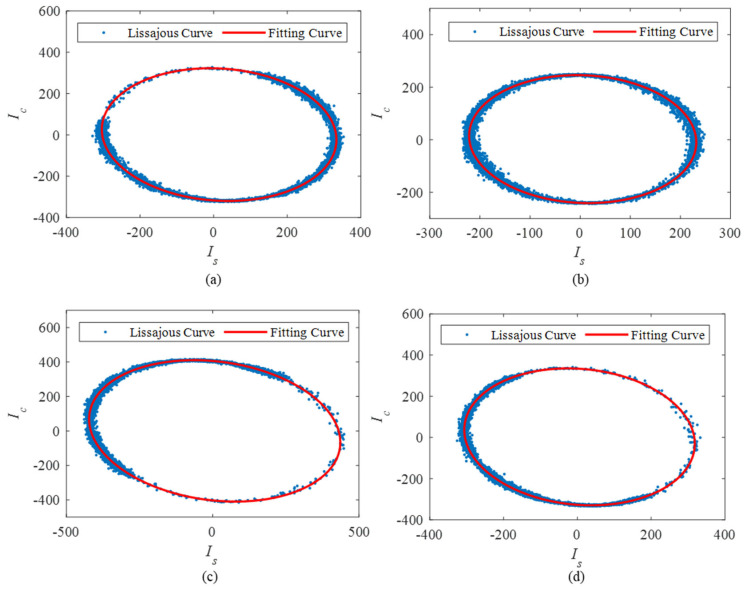
The Lissajous figures corresponding to the 4 sensors: (**a**) the Lissajous figures corresponding to S1; (**b**) the Lissajous figures corresponding to S2; (**c**) the Lissajous figures corresponding to S3; (**d**) the Lissajous figures corresponding to S4.

**Figure 17:**
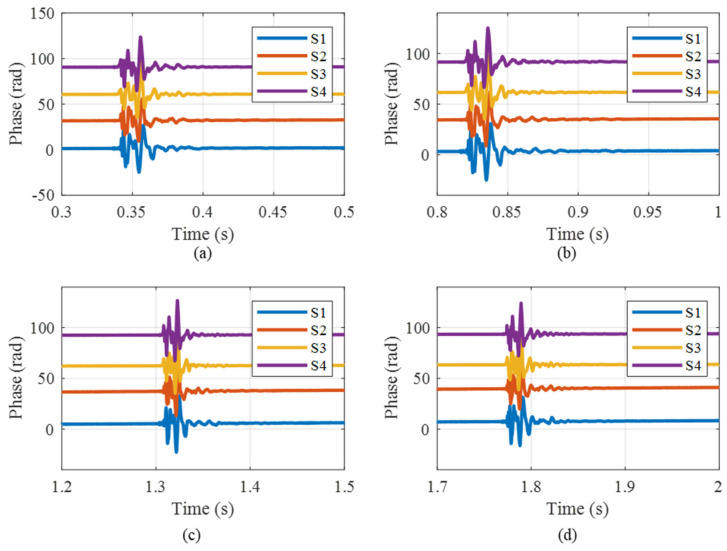
The calculated phase signals incorporating the calibration factor: (**a**) the calculated phase signals of the 4 sensors during the period from 0.3 s to 0.5 s; (**b**) the calculated phase signals of the 4 sensors during the period from 0.8 s to1 s; (**c**) the calculated phase signals of the 4 sensors during the period from 1.2 s to 1.5 s; (**d**) the calculated phase signals of the 4 sensors during the period from 1.7 s to 2 s.

According to the TDM and RPBPM technology, the three-step light intensity signals corresponding to the four sensors (S1, S2, S3 and S4 respectively) are shown in Figure 15 and the corresponding Lissajous figures are shown in Figure 16. It is observed that the fitting ellipses are close to but not a standard circle. The obtained correction factors are 1.08, 1.09, 0.84, 1.13, respectively. The calculated phase signals incorporating the calibration factors are shown in Figure 17. The vibration occurs during the time intervals of 0.3 s–0.4 s, 0.8 s–0.9 s, 1.3 s–1.4 s, and 1.7 s–1.8 s, which is consistent with the position of the abrupt change in vibration light intensities in Figure 15. Meanwhile, the findings indicate that the time and amplitude readings from the four sensors are consistent. Minor inaccuracies arise from variations in sensor sensitivity and placement. The experimental results effectively demonstrate the accuracy and adaptability of the RPBPM method.

## 8. Conclusions

In this study, an analysis of the two main error sources of the RPB method, i.e., the sampling interval and modulation depth deviation, were effectively carried out. Firstly, the error produced by the sampling interval was analyzed. The increase in sampling interval increased the time and frequency error. The sampling interval must be reasonably set according to the application requirements. Secondly, the error produced by the modulation depth deviation was analyzed, and the calibration of the modulation error was achieved based on the ellipse fitting of the Lissajous figure method. In practical applications, for a given system, the sampling interval and modulation depth are determined by the system parameters and typically remain fixed. Therefore, we can set and optimize them based on the above two points of analysis. When long-term signal acquisition is required, signal processing can be performed in segments. Finally, an accurate demodulation of the actual vibration signal was effectively achieved, which may effectively guide the practical application of the RPB method.

## Figures and Tables

**Table 1:** Simulation parameter settings.

Parameter	*D*	*f*	Δt	*A*	*B*	$\varphi_{0}$	*T*	ΔL
Value	10 rad	100 Hz	50 ns	0	1	0	1000 ns	10 m

**Table 2:** The parameter settings of the modulation depth deviation simulation.

Parameter	*D*	*f*	$\varphi_{0}$	δ
Value	10 rad	100 Hz	0	π/4

**Table 3:** The results of PGC demodulation method at different frequencies.

Signal Frequency (Hz)	RAD (%)	MHR (dB)	SINAD (dB)
100	0	96.84	96.83
500	1	64.8	64.7
1000	1.2	54.425	54.03
2000	0.3	21.68	20.87

**Table 4:** The results of heterodyne demodulation method at different frequencies.

Signal Frequency (Hz)	RAD (%)	MHR (dB)	SINAD (dB)
100	0	89.52	89.5
500	0.35	96.5	96.5
1000	0.22	81.23	81.23
2000	0.83	71.62	66.6
3000	0.48	30	30

**Table 5:** The results of RPBPM method at different frequencies.

Signal Frequency (Hz)	RAD (%)	MHR (dB)	SINAD (dB)
100	0.000026	80.81	79.37
500	0.00064	66.83	65.39
1000	0.0026	60.81	59.37
2000	0.010	54.78	53.35
3000	0.023	51.26	49.83

**Table 6:** The results of PGC demodulation method at different amplitudes.

Signal Amplitude (rad)	RAD (%)	MHR (dB)	SINAD (dB)
1	0.1	72.08	72.03
5	0.2	69.47	68.6
10	1.6	58.15	57.68
20	5.3	41.8	40.9
22	9	30.2	27.89

**Table 7:** The results of heterodyne demodulation method at different amplitudes.

Signal Amplitude (rad)	RAD (%)	MHR (dB)	SINAD (dB)
1	0.1	95.88	95.8
5	0.28	73.75	73.7
10	0.9	55.7	55.69
20	4.15	44.77	44.76
30	9.2	37.74	37.73

**Table 8:** The results of RPBPM method at different amplitudes.

Signal Amplitude (rad)	RAD (%)	MHR (dB)	SINAD (dB)
20	0.00051	67.27	61.13
30	0.00058	68.21	61.12
90	0.00049	71.03	61.08
100	0.00022	69.36	61.13
101	28	14.94	12.91

## Data Availability

The raw data supporting the conclusions of this article will be made available by the corresponding author on request.
